# Correction: On the Apportionment of Population Structure

**DOI:** 10.1371/journal.pone.0163343

**Published:** 2016-09-12

**Authors:** The PLOS ONE Staff

In the Author Contributions section, Omri Tal (OT) should also be listed under Wrote the paper, Wrote the Appendix and performed and analyzed the simulations, and Revision of manuscript.

The legend of Fig 11 appears incorrectly in the published article. The second item on the legend should read: Medium structure. Please see the correct Fig 11 and its caption here. The publisher apologizes for the error.

**Fig 11:**
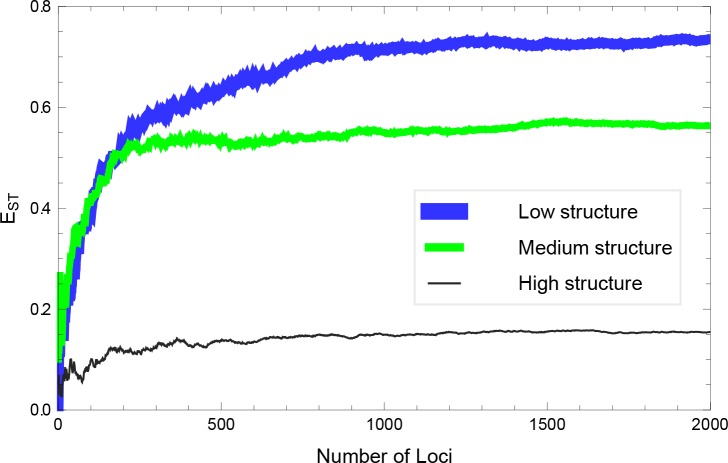
A numerical simulation of a model for *E*_ST_ for two structured populations (with *F*_ST_ = 0.05). *E*_ST_ was computed using the formulation in Eq (2) of *Materials and Methods*.
