# Supplementary figures and images for: On the Apportionment of Population Structure
Source: PLoS One. 2016 Aug 9;11(8):e0160413. doi: 10.1371/journal.pone.0160413 (PMC4978449; doi:10.1371/journal.pone.0160413)

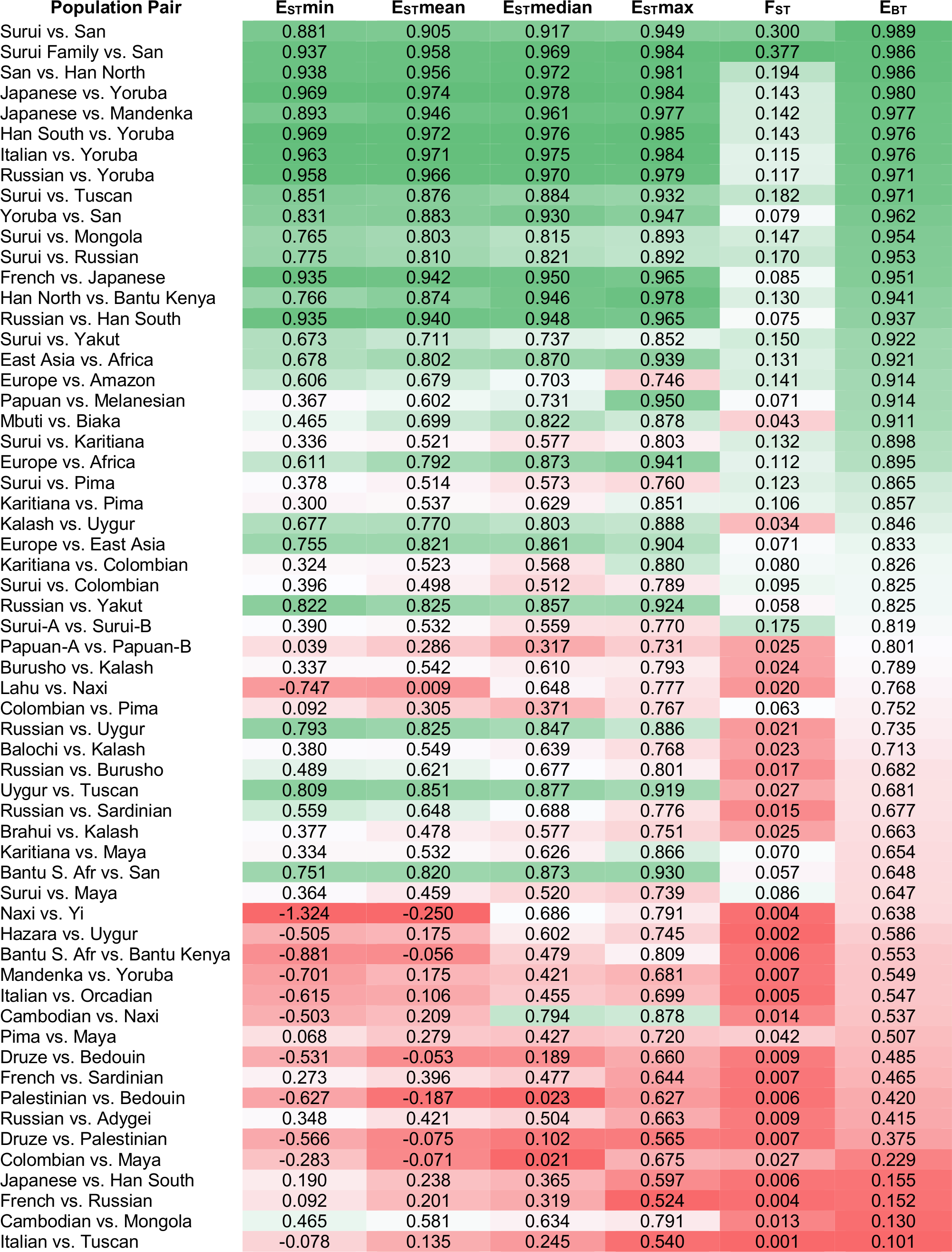

Supplement: S1 Table — (TIF) [file pone.0160413.s001.tif]

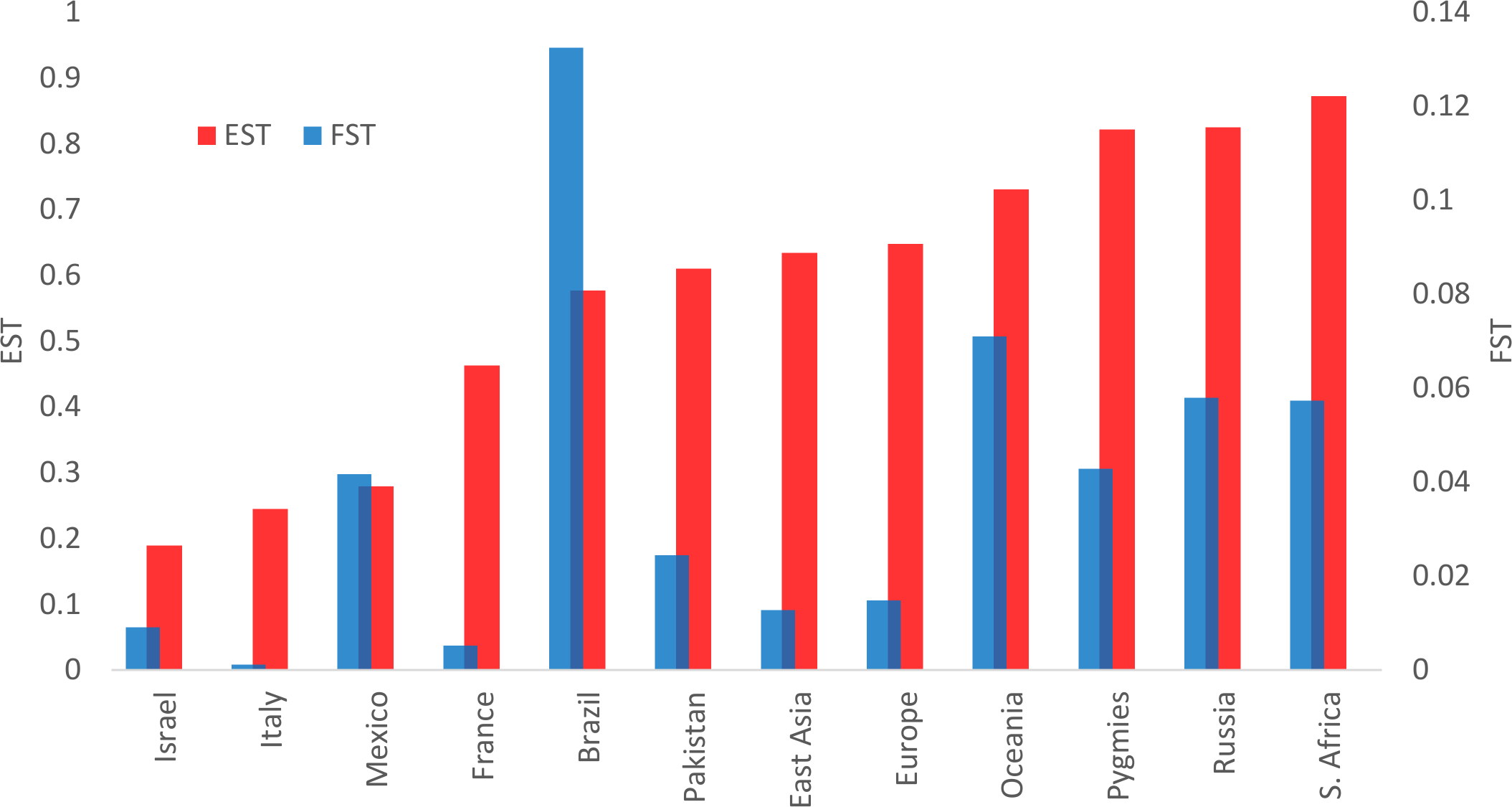

Supplement: S1 Fig — Calculated from a single pair of populations per region: Israel (Bedouins vs. Druze), Italy (North Italians vs. Tuscans), Mexico (Maya vs. Pima), France (Basque vs. French), Brazil (Karitiana vs. Surui), Pakistan (Burusho vs. Kalash) East Asia (Cambodian vs. Mongola), Europe (Russians vs. Sardinians), Oceania (Melanesians vs. Papuans), Pygmies (Biaka vs. Mbuti), Russia (Russians vs. Yakut) and Southern Africans (South African Bantu vs. San). The most obvious discrepancy between FST and EST is in Brazil, with a high FST and moderate EST. The Druze and Bedouin of Israel live within a few hundred km of each other, speak the same language, and have the lowest EST among these 12 pairs, yet have a somewhat higher FST (several times higher than between the two Italian populations from Northern Italy and Tuscany and almost twice as high as between the French and Basques). (TIF) [file pone.0160413.s002.tif]

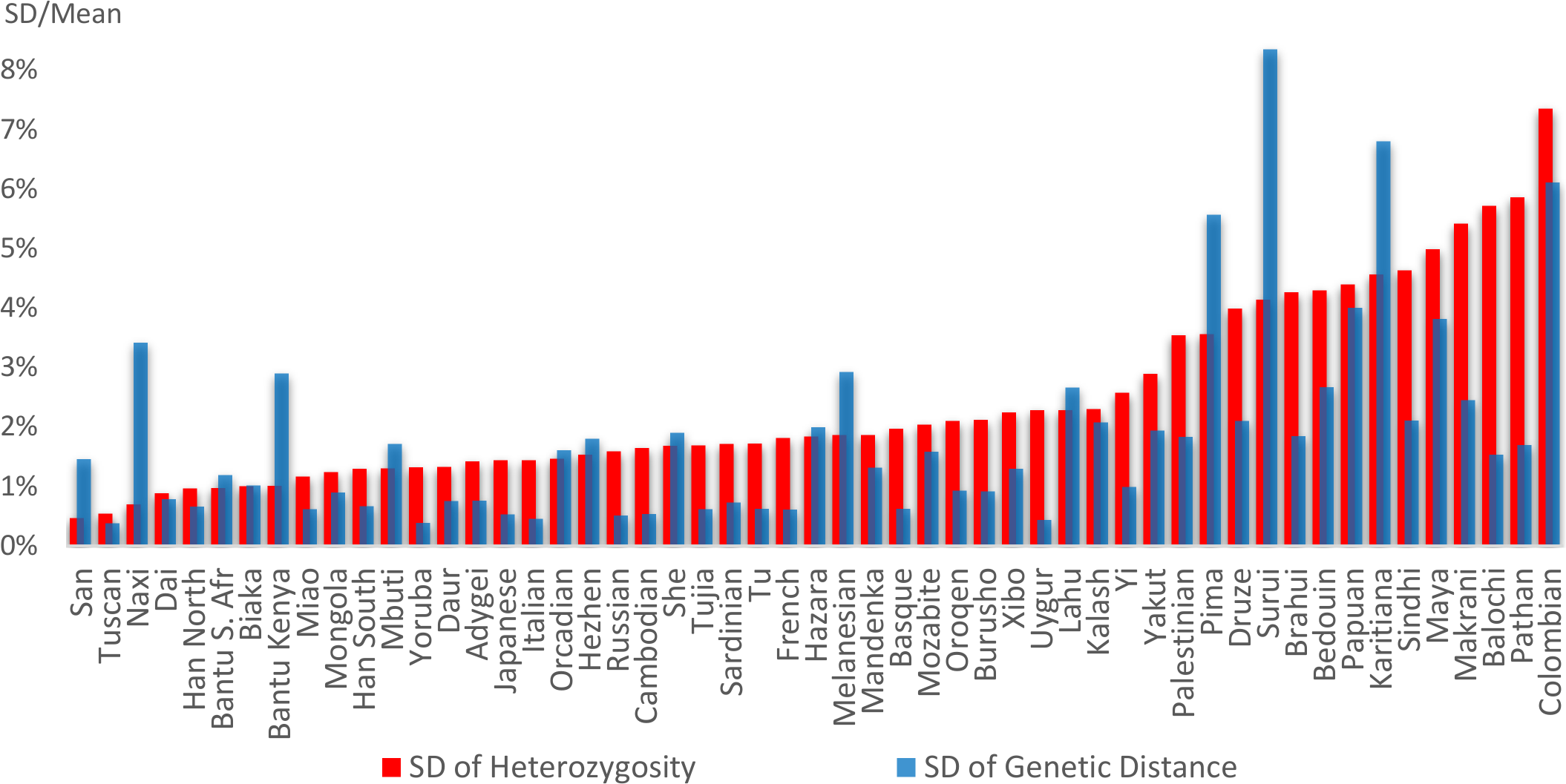

Supplement: S2 Fig — From 660,755 SNPs in 53 HGDP populations. Excessive SD of genetic distance (blue) compared to SD of heterozygosity (red), as in the San and Naxi samples, implies the inclusion of relatives. (TIF) [file pone.0160413.s003.tif]

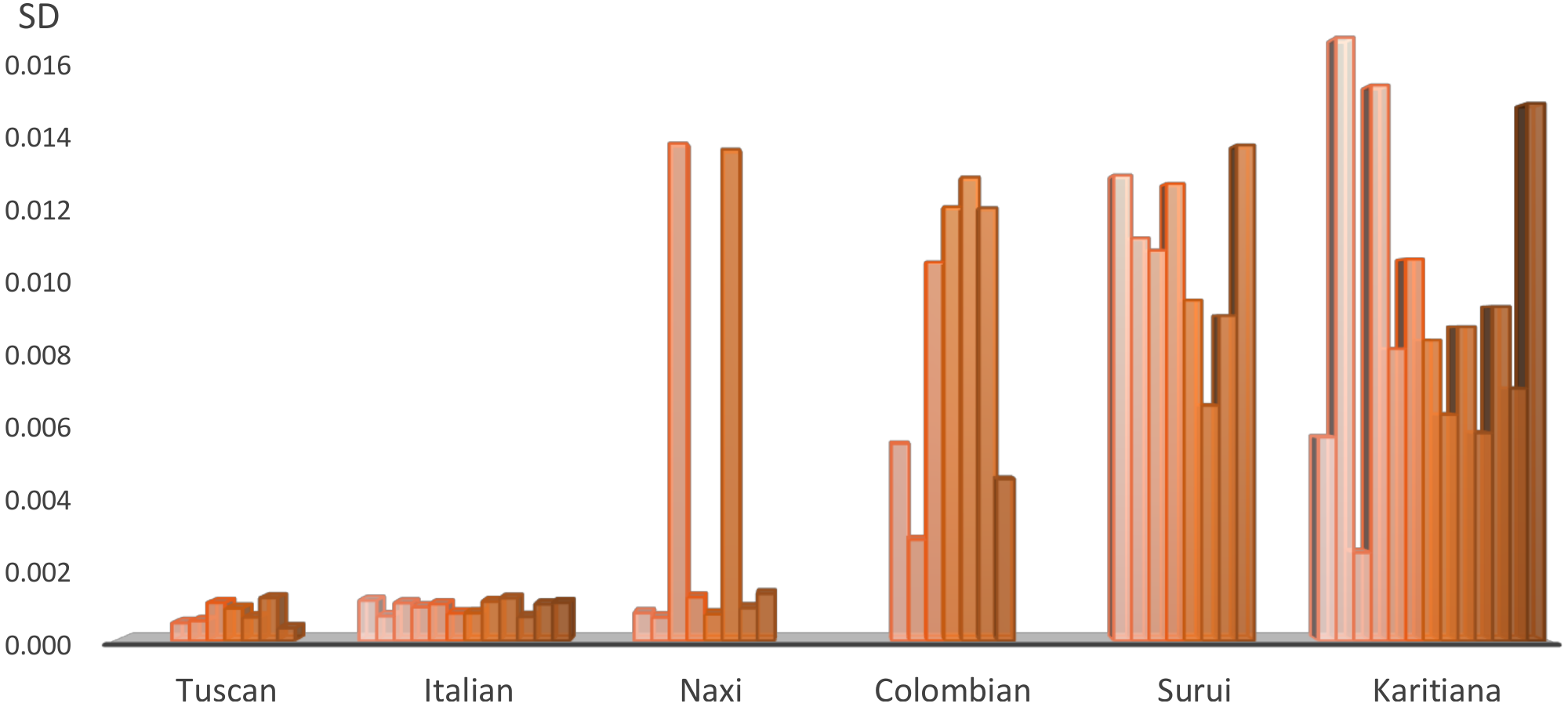

Supplement: S3 Fig — Each column represents the SD between a single individual and all other samples in the given population. Tuscans (n = 7), Italians (n = 12), Naxi (n = 8), Colombian (n = 7), Surui (n = 8), and Karitiana (n = 13). The “twin towers” in the Naxi batch are inferred to be a pair of close relatives in an otherwise panmictic population sample. These two individuals stick out like a sore thumb, while similarly related individuals are harder to identify among the Native American samples due to a higher base-level of structure in these population samples. (TIF) [file pone.0160413.s004.tif]

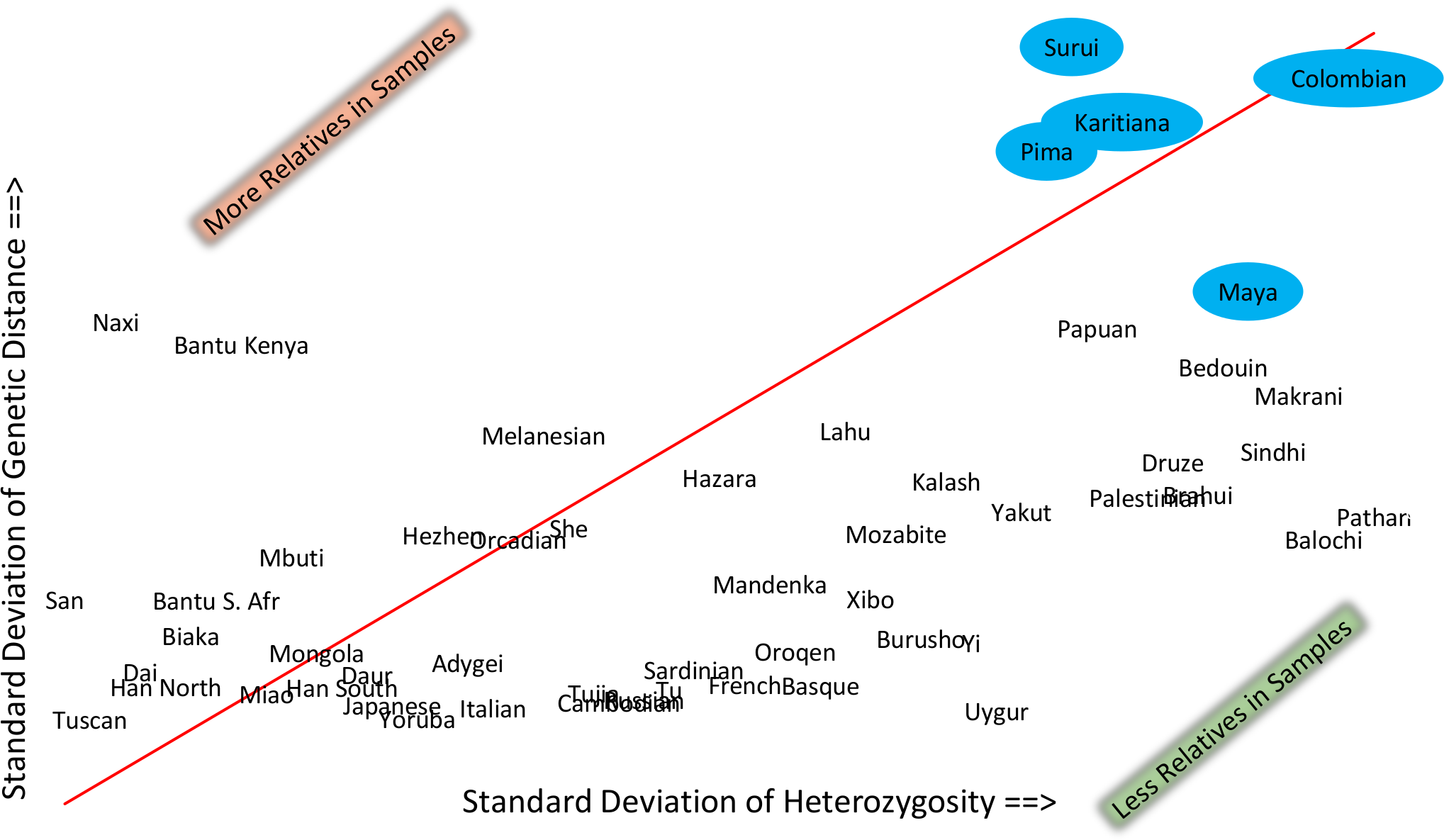

Supplement: S4 Fig — Generated from the entire HGDP dataset (938 individuals from 53 populations). The red diagonal line represents the linear trend line of the standard deviation of heterozygosity. Populations above this line are inferred to have more genetic structure than expected from heterozygosity, implying that relatives may have been included in the samples. Native American populations, highlighted in light blue, appear to have moderate or moderately high levels of relatives included among their samples. (TIF) [file pone.0160413.s005.tif]

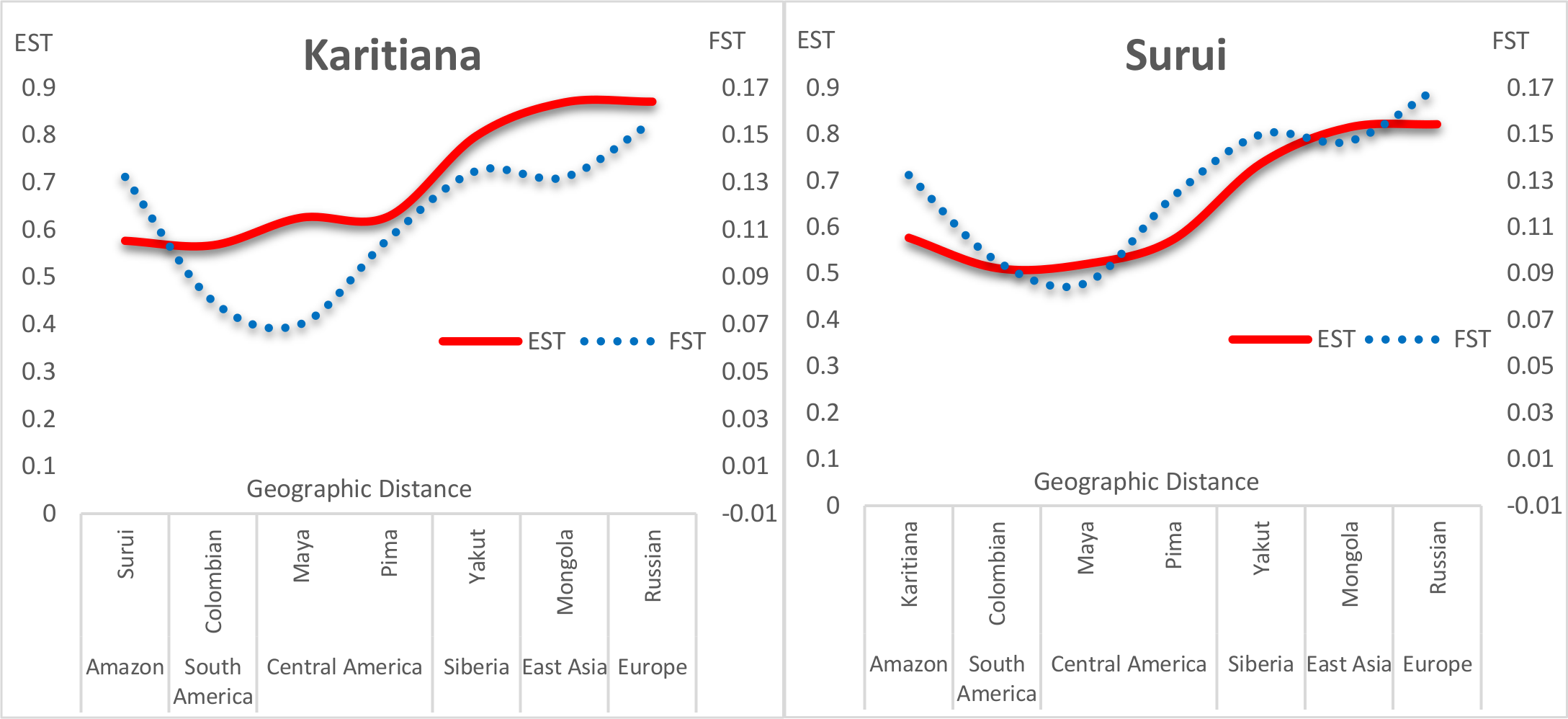

Supplement: S5 Fig — (TIF) [file pone.0160413.s006.tif]

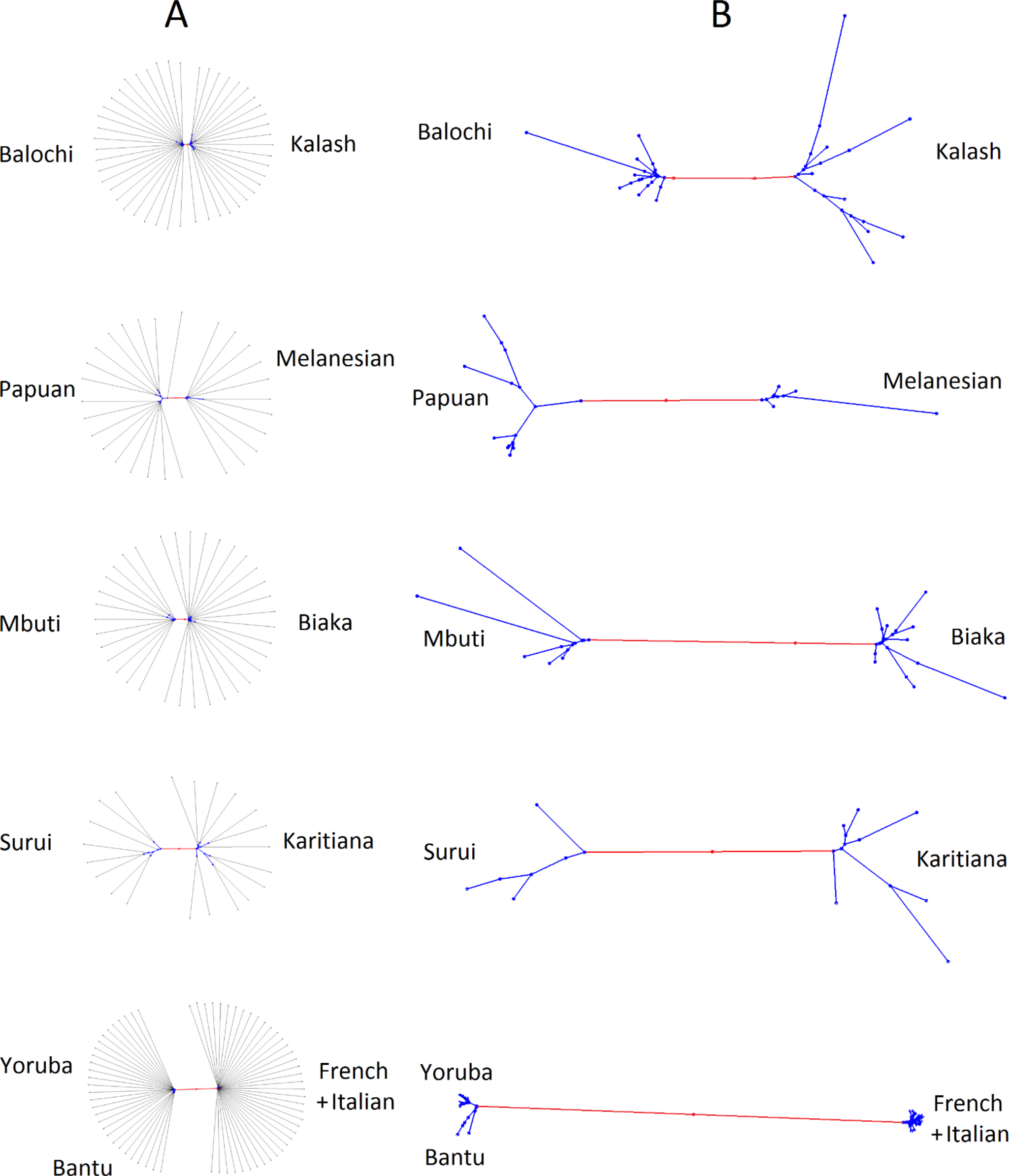

Supplement: S6 Fig — Generated from 660,755 SNPs. Individual branches are black, inter-population branches are red, and intra-population branches are blue. A. Complete trees. B. Zoom into trees with individual branches (black) removed. (TIF) [file pone.0160413.s007.tif]

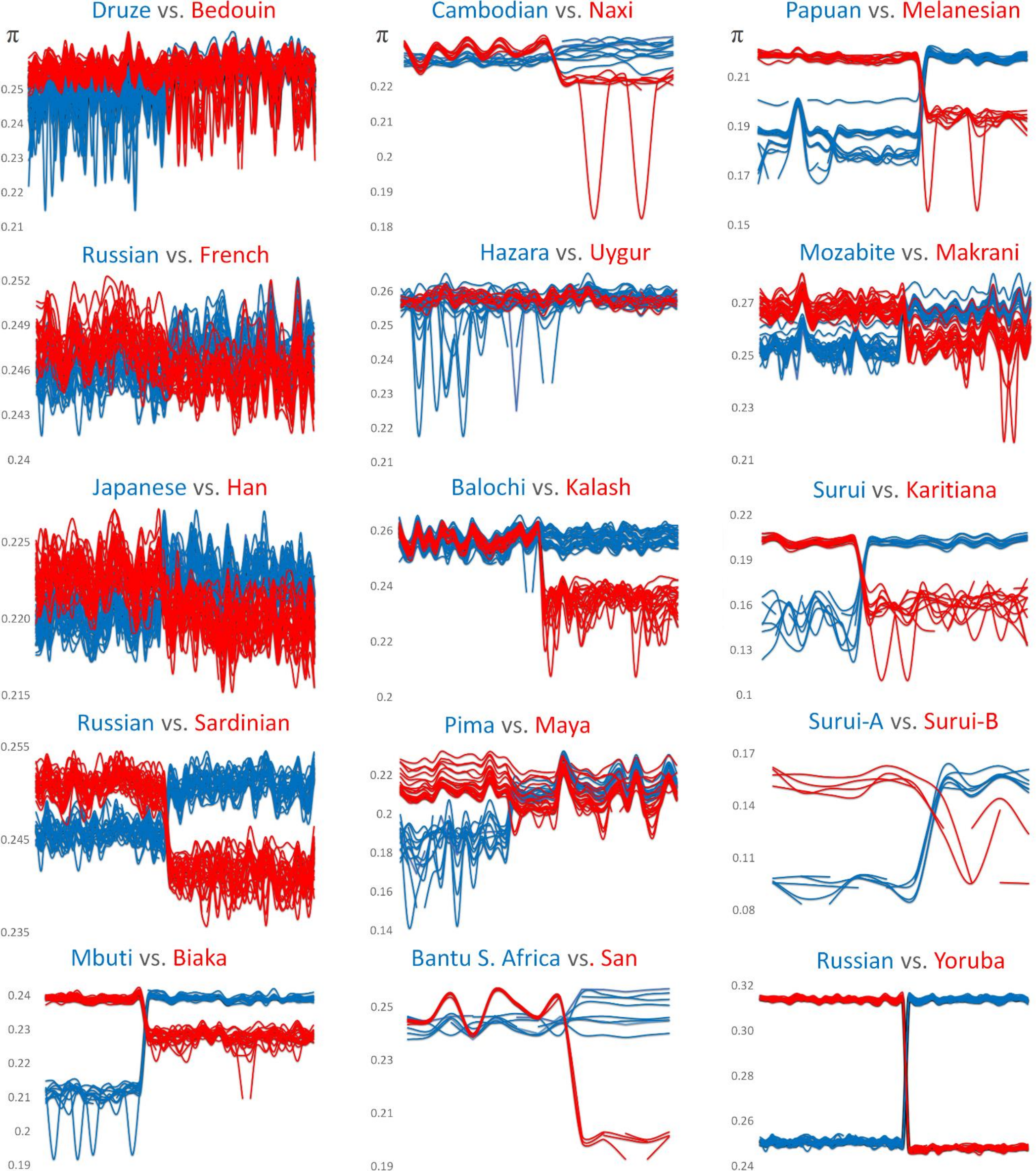

Supplement: S7 Fig — Each sample is represented by a red or blue string and each point on each string reflects distance between a pair of samples. Points that fall far below the rest are inferred to reflect close relatives. (TIF) [file pone.0160413.s008.tif]

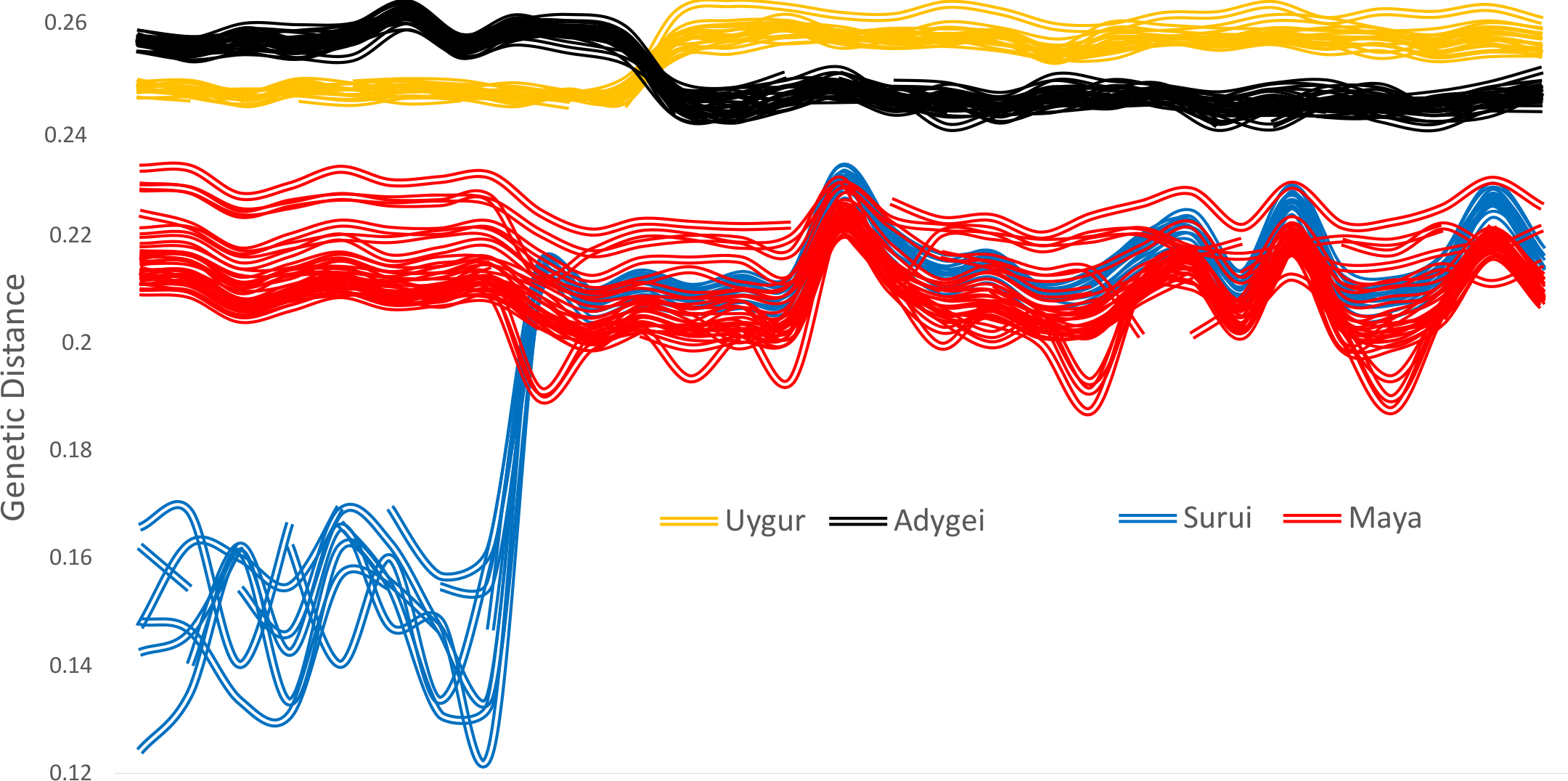

Supplement: S8 Fig — This is the same kind of plot as in S7 Fig, with each string representing a single individual. Despite a high FST of 0.09 (EST = 0.52), some Mayan individuals (red) are genetically closer to some Surui individuals (blue) than to some fellow Mayan individuals (ω > 0), presumably due to outbreeding (some Mayan individuals have significant European admixture, which increases distances among Mayans). There is no such overlap between Uygur (yellow) and Adygei (black) samples (ω = 0) despite a much lower pairwise FST of 0.02 (EST = 0.79). (TIF) [file pone.0160413.s009.tif]

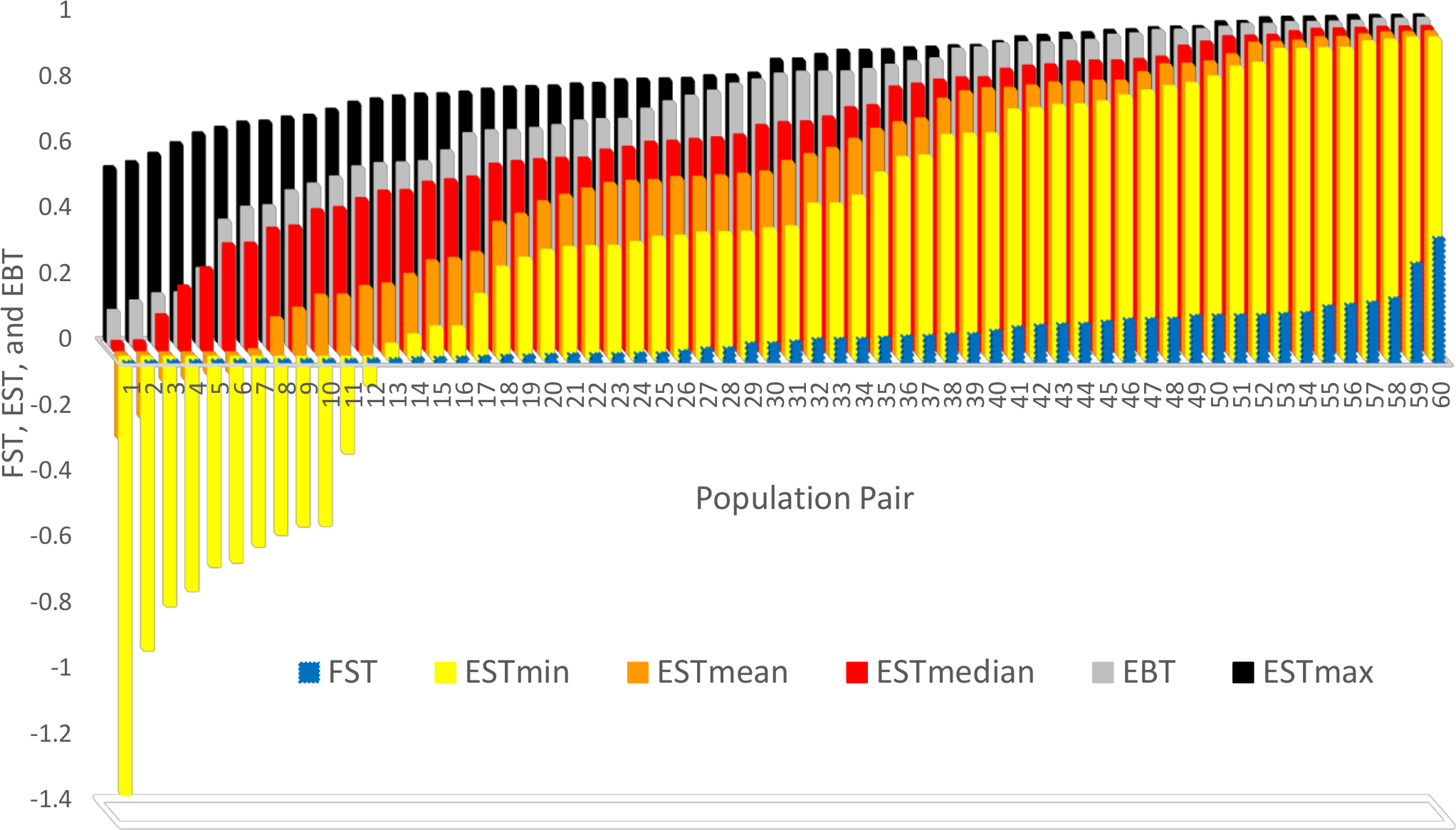

Supplement: S9 Fig — Negative ESTmin (yellow) and ESTmean (orange) would imply that close relatives were included among these samples. Of the 60 population pairs in the analysis, 12 (20%) have negative ESTmin values and 6 have negative ESTmean values. ESTmedian, ESTmax, and EBT cover virtually the entire 0–1 range with no negative values in these samples. The general trend is FST < ESTmin < ESTmean < ESTmedian < ESTmax. EBT (gray) is usually somewhere between ESTmedian (red) and ESTmax (black). (TIF) [file pone.0160413.s010.tif]

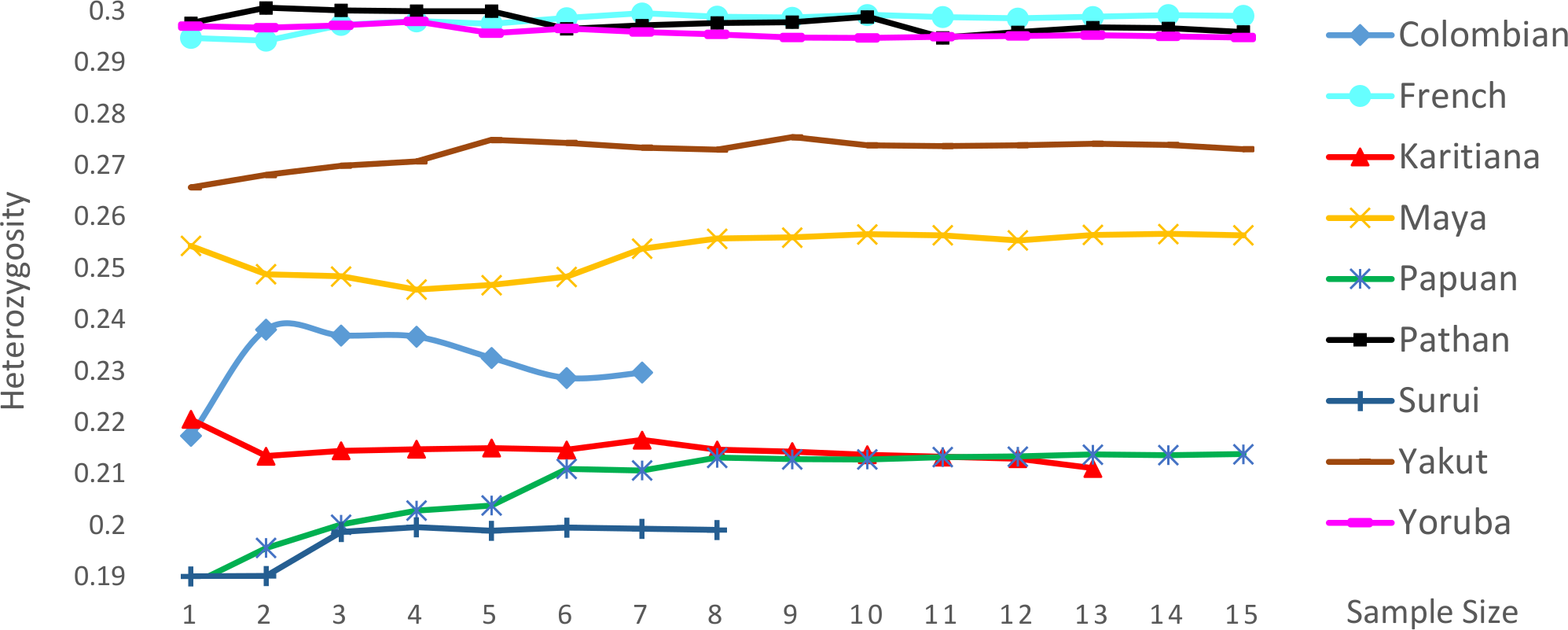

Supplement: S10 Fig — Heterozygosity in various HGDP populations with sample size increasing from 1 to 15. All samples were included in populations with less than 15 samples (namely 7 in Colombians, 8 in Surui, and 13 in Karitiana). (TIF) [file pone.0160413.s011.tif]

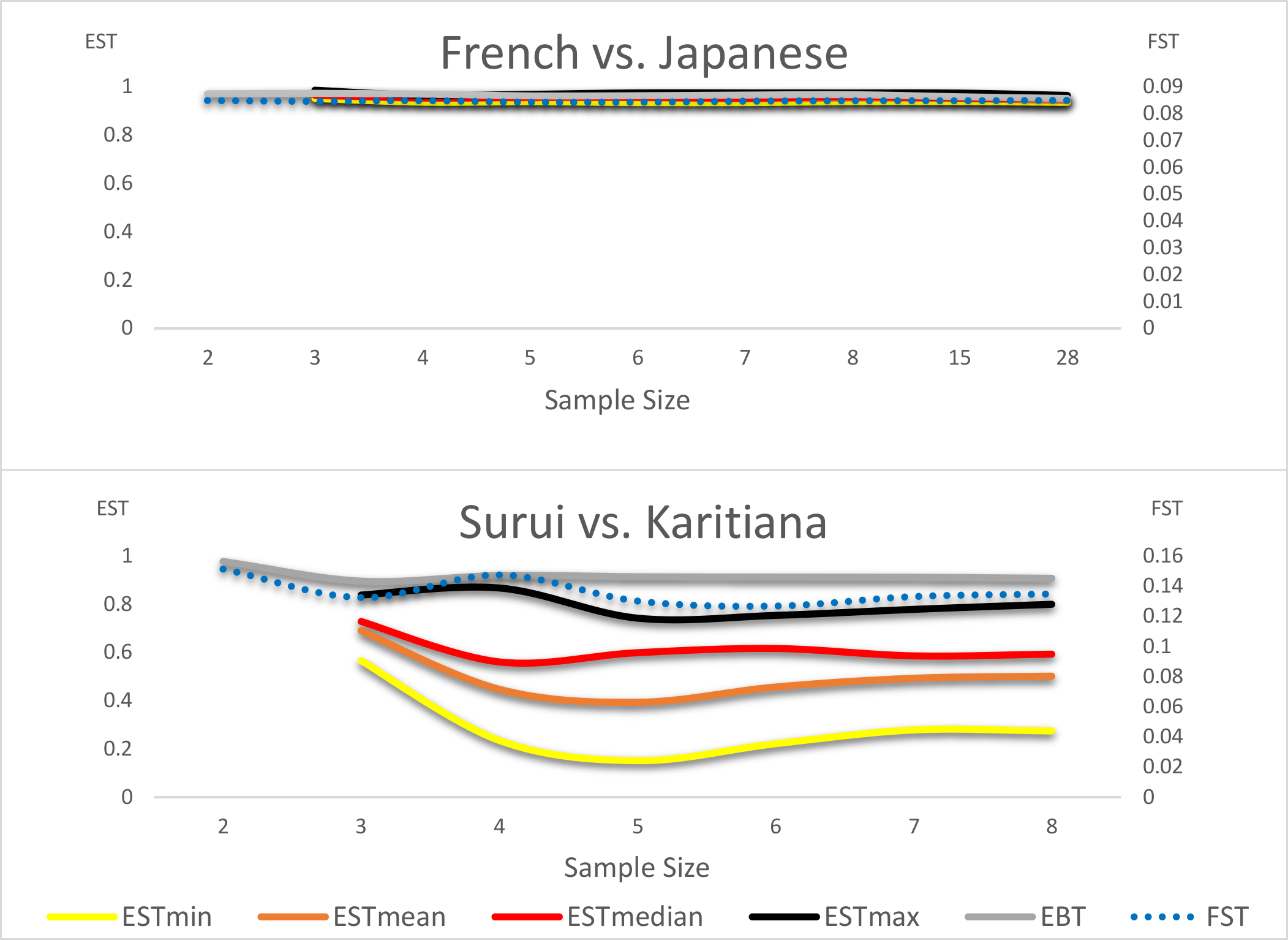

Supplement: S11 Fig — Differentiation was estimated in two population pairs: French-Japanese and Surui-Karitiana, with population sample sizes ranging from n = 2 to n = 8. French-Japanese estimates were also taken at n = 15 and n = 28 due to their larger samples. FST and EBT start at n = 2; EST starts at n = 3, the minimal sample size for estimating the standard deviation of pairwise distances. (TIF) [file pone.0160413.s012.tif]
